# Supplementary material for: Cooperative interactions facilitate stimulation of Rad51 by the Swi5-Sfr1 auxiliary factor complex
Source: eLife. 2020 Mar 24;9:e52566. doi: 10.7554/eLife.52566 (PMC7093153; doi:10.7554/eLife.52566)
Supplement: Supplementary file 1. [file elife-52566-supp1.docx]

**Supplementary File 1**

| **Key Resources Table** | | | | |
| --- | --- | --- | --- | --- |
| **Reagent type (species) or resource** | **Designation** | **Source or reference** | **Identifiers** | **Additional information** |
| strain, strain background (Schizosaccharomyces pombe; *Msmt-0 leu-1-32 ura4-D18 his3-D1 isp6::hphMX4 psp3::kanMX4*) | Rad55-Rad57 overproducer | Hiroshi Iwasaki | BA1 | Protease-deficient strain used to overproduce and partially purify Rad55-Rad57. |
| strain, strain background (S. pombe; *Msmt-0 leu-1-32 ura4-D18 his3-D1 arg3-D1*) | *sfr1-3A* | This work | BA9 | See Materials and Methods, *S. pombe* strains |
| strain, strain background (S. pombe; *Msmt-0 leu-1-32 ura4-D18 his3-D1 arg3-D1*) | *sfr1-4A* | This work | BA10 | See Materials and Methods, *S. pombe* strains |
| strain, strain background (S. pombe; *Msmt-0 leu-1-32 ura4-D18 his3-D1 arg3-D1*) | *sfr1-7A* | This work | BA12 | See Materials and Methods, *S. pombe* strains |
| strain, strain background (S. pombe; *Msmt-0 leu-1-32 ura4-D18 his3-D1 arg3-D1*) | *rad51::his3^+^* | Tsutsui et al. 2000 | BA29 | See Materials and Methods, *S. pombe* strains |
| strain, strain background (S. pombe; *Msmt-0 leu-1-32 ura4-D18 his3-D1 arg3-D1*) | *rad57::arg3^+^* | Hiroshi Iwasaki | BA31 | See Materials and Methods, *S. pombe* strains |
| strain, strain background (S. pombe; *Msmt-0 leu-1-32 ura4-D18 his3-D1 arg3-D1*) | *rad55::arg3^+^* | Tsutsui et al. 2001 | BA51 | See Materials and Methods, *S. pombe* strains |
| strain, strain background (S. See Materials and Methods, *S. pombe* strains pombe; *Msmt-0 leu-1-32 ura4-D18 his3-D1 arg3-D1*) | *Wild type* | Akamatsu et al. 2003 | BA53 | See Materials and Methods, *S. pombe* strains |
| strain, strain background (S. pombe; *Msmt-0 leu-1-32 ura4-D18 his3-D1 arg3-D1*) | *rad55::arg3^+^ sfr1-7A* | This work | BA109 | See Materials and Methods, *S. pombe* strains |
| strain, strain background (S. pombe; *Msmt-0 leu-1-32 ura4-D18 his3-D1 arg3-D1*) | *rad57::arg3^+^ sfr1-7A* | This work | BA111 | See Materials and Methods, *S. pombe* strains |
| strain, strain background (S. pombe; *Msmt-0 leu-1-32 ura4-D18 his3-D1 arg3-D1*) | *rad57::arg3^+^ sfr1-3A* | This work | BA116 | See Materials and Methods, *S. pombe* strains |
| strain, strain background (S. pombe; *Msmt-0 leu-1-32 ura4-D18 his3-D1 arg3-D1*) | *rad55::arg3^+^ sfr1-4A* | This work | BA118 | See Materials and Methods, *S. pombe* strains |
| strain, strain background (S. pombe; *Msmt-0 leu-1-32 ura4-D18 his3-D1 arg3-D1*) | *rad57::arg3^+^ sfr1-4A* | This work | BA120 | See Materials and Methods, *S. pombe* strains |
| strain, strain background (S. pombe; *Msmt-0 leu-1-32 ura4-D18 his3-D1 arg3-D1*) | *rad55::arg3^+^ sfr1-3A* | This work | BA122 | See Materials and Methods, *S. pombe* strains |
| strain, strain background (S. pombe; *Msmt-0 leu-1-32 ura4-D18 his3-D1 arg3-D1*) | *sfr1::kanMX6* | This work | BA125 | See Materials and Methods, *S. pombe* strains |
| strain, strain background (S. pombe; *Msmt-0 leu-1-32 ura4-D18 his3-D1 arg3-D1*) | *rad55::arg3^+^ sfr1::kanMX6* | This work | BA126 | See Materials and Methods, *S. pombe* strains |
| strain, strain background (S. pombe; *Msmt-0 leu-1-32 ura4-D18 his3-D1 arg3-D1*) | *rad57::arg3^+^ sfr1::kanMX6* | This work | BA130 | See Materials and Methods, *S. pombe* strains |
| strain, strain background (S. pombe; *Msmt-0 leu-1-32 ura4-D18 his3-D1 arg3-D1*) | *rad55::natMX6* | This work | BA150 | See Materials and Methods, *S. pombe* strains |
| strain, strain background (S. pombe; *Msmt-0 leu-1-32 ura4-D18 his3-D1 arg3-D1*) | *sfr1N* | This work | BA166 | See Materials and Methods, *S. pombe* strains |
| strain, strain background (S. pombe; *Msmt-0 leu-1-32 ura4-D18 his3-D1 arg3-D1*) | *sfr1C* | This work | BA168 | See Materials and Methods, *S. pombe* strains |
| strain, strain background (S. pombe; *Msmt-0 leu-1-32 ura4-D18 his3-D1 arg3-D1*) | *rad55::natMX6 sfr1N* | This work | BA178 | See Materials and Methods, *S. pombe* strains |
| strain, strain background (S. pombe; *Msmt-0 leu-1-32 ura4-D18 his3-D1 arg3-D1*) | *rad55::natMX6 sfr1C* | This work | BA182 | See Materials and Methods, *S. pombe* strains |
| strain, strain background (S. pombe; *Msmt-0 leu-1-32 ura4-D18 his3-D1 arg3-D1*) | *7MYC-sfr1-kanMX6* | This work | BA204 | See Materials and Methods, *S. pombe* strains |
| strain, strain background (S. pombe; *Msmt-0 leu-1-32 ura4-D18 his3-D1 arg3-D1*) | *7MYC-sfr1-7A-kanMX6* | This work | BA244 | See Materials and Methods, *S. pombe* strains |
| strain, strain background (S. pombe; *Msmt-0 leu-1-32 ura4-D18 his3-D1 arg3-D1*) | *NLS-sfr1N-kanMX6* | This work | BA310 | See Materials and Methods, *S. pombe* strains |
| strain, strain background (S. pombe; *Msmt-0 leu-1-32 ura4-D18 his3-D1 arg3-D1*) | *NLS-sfr1C-kanMX6* | This work | BA311 | See Materials and Methods, *S. pombe* strains |
| strain, strain background (S. pombe; *Msmt-0 leu-1-32 ura4-D18 his3-D1 arg3-D1*) | *7MYC-sfr1N-kanMX6* | This work | BA314 | See Materials and Methods, *S. pombe* strains |
| strain, strain background (S. pombe; *Msmt-0 leu-1-32 ura4-D18 his3-D1 arg3-D1*) | *7MYC-sfr1C-kanMX6* | This work | BA316 | See Materials and Methods, *S. pombe* strains |
| strain, strain background (*Escherichia coli, BL21 [DE3]*) | Sfr1N / Rad51  - / + | This work | BA009 | See Materials and Methods, *E. coli* strains |
| strain, strain background (*Escherichia coli, BL21 [DE3]*) | Sfr1N / Rad51  - / - | This work | BA0058 | See Materials and Methods, *E. coli* strains |
| strain, strain background (*Escherichia coli, BL21 [DE3]*) | Sfr1N / Rad51  + / - | This work | BA0060 | See Materials and Methods, *E. coli* strains |
| strain, strain background (*Escherichia coli, BL21 [DE3]*) | Sfr1N / Rad51  + / + | This work | BA0011 | See Materials and Methods, *E. coli* strains |
| strain, strain background (*Escherichia coli, BL21 [DE3]*) | Sfr1N / Rad51  K93 / + | This work | BA0013 | See Materials and Methods, *E. coli* strains |
| strain, strain background (*Escherichia coli, BL21 [DE3]*) | Sfr1N / Rad51  K94 / + | This work | BA0015 | See Materials and Methods, *E. coli* strains |
| strain, strain background (*Escherichia coli, BL21 [DE3]*) | Sfr1N / Rad51  R95 / + | This work | BA0017 | See Materials and Methods, *E. coli* strains |
| strain, strain background (*Escherichia coli, BL21 [DE3]*) | Sfr1N / Rad51  A96 / + | This work | BA0019 | See Materials and Methods, *E. coli* strains |
| strain, strain background (*Escherichia coli, BL21 [DE3]*) | Sfr1N / Rad51  R97 / + | This work | BA0021 | See Materials and Methods, *E. coli* strains |
| strain, strain background (*Escherichia coli, BL21 [DE3]*) | Sfr1N / Rad51  E98 / + | This work | BA0023 | See Materials and Methods, *E. coli* strains |
| strain, strain background (*Escherichia coli, BL21 [DE3]*) | Sfr1N / Rad51  A99 / + | This work | BA0025 | See Materials and Methods, *E. coli* strains |
| strain, strain background (*Escherichia coli, BL21 [DE3]*) | Sfr1N / Rad51  K100 / + | This work | BA0027 | See Materials and Methods, *E. coli* strains |
| strain, strain background (*Escherichia coli, BL21 [DE3]*) | Sfr1N / Rad51  N101 / + | This work | BA0029 | See Materials and Methods, *E. coli* strains |
| strain, strain background (*Escherichia coli, BL21 [DE3]*) | Sfr1N / Rad51  I102 / + | This work | BA0031 | See Materials and Methods, *E. coli* strains |
| strain, strain background (*Escherichia coli, BL21 [DE3]*) | Sfr1N / Rad51  L103 / + | This work | BA0033 | See Materials and Methods, *E. coli* strains |
| strain, strain background (*Escherichia coli, BL21 [DE3]*) | Sfr1N / Rad51  L104 / + | This work | BA0035 | See Materials and Methods, *E. coli* strains |
| strain, strain background (*Escherichia coli, BL21 [DE3]*) | Sfr1N / Rad51  K105 / + | This work | BA0037 | See Materials and Methods, *E. coli* strains |
| strain, strain background (*Escherichia coli, BL21 [DE3]*) | Sfr1N / Rad51  P106 / + | This work | BA0039 | See Materials and Methods, *E. coli* strains |
| strain, strain background (*Escherichia coli, BL21 [DE3]*) | Sfr1N / Rad51  F107 / + | This work | BA0044 | See Materials and Methods, *E. coli* strains |
| strain, strain background (*Escherichia coli, BL21 [DE3]*) | Sfr1N / Rad51  K108 / + | This work | BA0046 | See Materials and Methods, *E. coli* strains |
| strain, strain background (*Escherichia coli, BL21 [DE3]*) | Sfr1N / Rad51  S109 / + | This work | BA0048 | See Materials and Methods, *E. coli* strains |
| strain, strain background (*Escherichia coli, BL21 [DE3]*) | Sfr1N / Rad51  P110 / + | This work | BA0050 | See Materials and Methods, *E. coli* strains |
| strain, strain background (*Escherichia coli, BL21 [DE3]*) | Sfr1N / Rad51  L111 / + | This work | BA0052 | See Materials and Methods, *E. coli* strains |
| strain, strain background (*Escherichia coli, BL21 [DE3]*) | Sfr1N / Rad51  R112 / + | This work | BA0054 | See Materials and Methods, *E. coli* strains |
| strain, strain background (*Escherichia coli, BL21 [DE3]*) | Sfr1N / Rad51  Q113 / + | This work | BA0056 | See Materials and Methods, *E. coli* strains |
| strain, strain background (*Escherichia coli, BL21 [DE3]*) | Sfr1N / Rad51  K157 / + | This work | BA0062 | See Materials and Methods, *E. coli* strains |
| strain, strain background (*Escherichia coli, BL21 [DE3]*) | Sfr1N / Rad51  R158 / + | This work | BA0064 | See Materials and Methods, *E. coli* strains |
| strain, strain background (*Escherichia coli, BL21 [DE3]*) | Sfr1N / Rad51  Q159 / + | This work | BA0066 | See Materials and Methods, *E. coli* strains |
| strain, strain background (*Escherichia coli, BL21 [DE3]*) | Sfr1N / Rad51  K160 / + | This work | BA0068 | See Materials and Methods, *E. coli* strains |
| strain, strain background (*Escherichia coli, BL21 [DE3]*) | Sfr1N / Rad51  R161 / + | This work | BA0070 | See Materials and Methods, *E. coli* strains |
| strain, strain background (*Escherichia coli, BL21 [DE3]*) | Sfr1N / Rad51  L162 / + | This work | BA0072 | See Materials and Methods, *E. coli* strains |
| strain, strain background (*Escherichia coli, BL21 [DE3]*) | Sfr1N / Rad51  F163 / + | This work | BA0074 | See Materials and Methods, *E. coli* strains |
| strain, strain background (*Escherichia coli, BL21 [DE3]*) | Sfr1N / Rad51  K164 / + | This work | BA0076 | See Materials and Methods, *E. coli* strains |
| strain, strain background (*Escherichia coli, BL21 [DE3]*) | Sfr1N / Rad51  S165 / + | This work | BA0078 | See Materials and Methods, *E. coli* strains |
| strain, strain background (*Escherichia coli, BL21 [DE3]*) | Sfr1N / Rad51  P166 / + | This work | BA0080 | See Materials and Methods, *E. coli* strains |
| Antibody | Rabbit monoclonal anti-MYC | Sigma-Aldrich | C3956 | 1:1,000 |
| Antibody | Rat polyclonal anti-Rad51 | Hiroshi Iwasaki |  | 1:10,000 |
| Antibody | Mouse monoclonal anti-tubulin | Sigma-Aldrich | T5168 | 1:10,000 |
| Antibody | Rabbit polyclonal anti-Sfr1 | Haruta et al. 2006 |  | 1:5,000 |
| Antibody | Rabbit polyclonal anti-Rad51 | Akamatsu et al. 2003 |  | 1:10,000 |
| Antibody | Mouse monoclonal anti-Sfr1 | Kokabu et al. 2011 | 1-5; 76-80 | 1:1,000 each |
| Antibody | Rabbit polyclonal anti-Rad55 | Hiroshi Iwasaki |  | 1:5,000 |
| Antibody | Rabbit polyclonal anti-Rad57 | Tsutsui et al. 2001 |  | 1:5,000 |
| Antibody | Anti-mouse IgG (HRP-conjugated) | GE Healthcare | NA931 | 1:5,000 |
| Antibody | Anti-rabbit IgG (HRP-conjugated) | GE Healthcare | NA934 | 1:5,000 |
| Antibody | Anti-rat IgG (HRP-conjugated) | Jackson Laboratories | 712-035-153 | 1:10,000 |
| recombinant DNA reagent | pEVOL-pBpF (plasmid) | Young et al. 2010 |  |  |
| recombinant DNA reagent | Poly-dT 72-mer with 5’ TAMRA label (oligonucleotide) | Eurofins Genomics |  | Used for fluorescence anisotropy assay in Figure 6B-E. |
| recombinant DNA reagent | PhiX174 virion DNA (ssDNA plasmid) | NEB | N3023L |  |
| recombinant DNA reagent | PhiX174 RF I DNA (dsDNA plasmid) | NEB | N3021L |  |
| peptide, recombinant protein | *S. pombe* Rad51 | Kurokawa et al. 2008 |  |  |
| peptide, recombinant protein | *S. pombe* RPA | Haruta et al. 2006 |  |  |
| peptide, recombinant protein | *S. pombe* Swi5-Sfr1 | Haruta et al. 2006 |  |  |
| peptide, recombinant protein | *S. pombe* Sfr1N | Kuwabara et al. 2010 |  |  |
| peptide, recombinant protein | *S. pombe* Swi5-Sfr1C | Kuwabara et al. 2010 |  |  |
| peptide, recombinant protein | *S. pombe* Swi5-Sfr1-7A | This study |  | See Materials and Methods, Purification of proteins for biochemical analysis |
| peptide, recombinant protein | *S. pombe* Swi5-Sfr1-3A | This study |  | See Materials and Methods, Purification of proteins for biochemical analysis |
| peptide, recombinant protein | *S. pombe* Swi5-Sfr1-4A | This study |  | See Materials and Methods, Purification of proteins for biochemical analysis |
| commercial assay or kit | Affi-gel 15 | Bio-Rad | 1536051 |  |
| commercial assay or kit | Amicon Ultra-15, 10K MWCO | Merck | UFC901096 |  |
| commercial assay or kit | Amylose resin | NEB | E8021L |  |
| commercial assay or kit | Dynabeads Protein A | ThermoFisher | 10002D |  |
| commercial assay or kit | ECL Prime Western Blotting Detection Reagent | GE Healthcare | RPN2232 |  |
| commercial assay or kit | Human IgG-Agarose | Sigma-Aldrich | A6284-5ML |  |
| commercial assay or kit | Malachite Green Phosphate Assay Kit | BioAssay Systems | POMG-25H |  |
| commercial assay or kit | PreScission Protease | GE Healthcare | 27084301 |  |
| commercial assay or kit | Sep-Pak C8 Plus Short Cartridge | Waters | WAT036775 |  |
| commercial assay or kit | UV lamp | UVP | B-100AP |  |
| chemical compound, drug | Phosphocreatine di(tris) salt | Sigma-Aldrich | P1937 |  |
| chemical compound, drug | ATP | Sigma-Aldrich | A2383 |  |
| chemical compound, drug | Bio-Safe Coomassie Stain | Bio-Rad | 1610786 |  |
| chemical compound, drug | SYBR Gold Nucleic Acid Gel Stain | ThermoFisher | S11494 |  |
| chemical compound, drug | Proteinase K | TaKaRa | 9034 |  |
| chemical compound, drug | Creatine Kinase | Sigma-Aldrich | 10127566001 |  |
| chemical compound, drug | H-p-Bz-Phe-OH (p-benzoyl-L-phenlyalanine, pBPA) | BACHEM | 40176460001 |  |
| chemical compound, drug | cOmplete protease inhibitor cocktail | Roche | 11836145001 |  |
| software, algorithm | MultiGauge version 3.2 | Fuji Film |  |  |
| software, algorithm | KalediaGraph | Synergy Software |  |  |
| software, algorithm | Prism version 7.0 | GraphPad |  |  |
